# Is it healthy urban agriculture? Human exposure to potentially toxic elements in urban gardens from Andalusia, Spain

**DOI:** 10.1007/s11356-024-33500-w

**Published:** 2024-05-15

**Authors:** Sabina Rossini-Oliva, Rafael López Nuñez

**Affiliations:** 1 Department of Plant Biology and Ecology, University of Seville, Avda. Reina Mercedes S/N, 41080 Seville, Spain; 2 Instituto de Recursos Naturales y Agrobiología de Sevilla (IRNAS-CSIC), Avda. Reina Mercedes 10, 41012 Seville, Spain

**Keywords:** Health risks, Metals, Pollution, Vegetables, Toxicity

## Abstract

Different vegetable species and topsoils were collected from different urban gardens of Seville, Cordoba, and Huelva (South Spain) and from two small towns in a mining area (Riotinto), together with topsoil close to the plants. The concentration of potentially toxic elements (PTEs) (As, B, Ba, Cd, Co, Cr, Cu, Mo, Ni, Pb, and Zn) was evaluated in edible plant parts and in the soils. The same species were also purchased from Seville local markets and from a peri-urban area (domestic garden in a rural area) and also analyzed. Plant/soil pollution relation was studied and human health risk was assessed by different parameters. Soils of urban gardens from the mining area were more contaminated with As, Cr, Cu, Pb, and Zn in comparison with other locations, and generally, soils from cities showed higher values of As, Pb, and Zn than the peri-urban ones. The mean concentration of almost all potentially toxic elements was higher in leafy than in fruiting and bulbous species. Arsenic, Cd, and Pb concentrations were below health-based guidance values in all vegetables except Cd in one sample in the peri-urban area. In general, PTEs concentration in vegetables from city urban gardens did not exceed the one found in market vegetables for almost all studied elements, except in lettuce for almost elements. The hazard quotient (HQ) values were lower than the unit for all PTEs in plant species from the studied gardens, as well as the hazard index (HI), indicating that consumption of these vegetables can be considered safe and without risk to human health. Also, cancer risk values for As were below the established limits in all vegetables from the studied urban gardens, including those from the As-contaminated soils in the mining area.

**Supplementary Information:**

The online version contains supplementary material available at 10.1007/s11356-024-33500-w.

## Introduction

Health is a matter of particular importance within the 2030 Agenda. The Sustainable Development Agenda 2030 delivered by the United Nations includes a target (SDG3, 3.9) that promotes the reduction of deaths and diseases caused by contamination (from hazardous chemicals, air, water, and soil). Anthropogenic activities contribute to increasing soil and air contamination in major cities and agricultural areas (Gaspéri et al. 2016; Wang et al. 2016; Cetin et al. 2022). Plants take up elements by air and soil. Therefore, it is essential to monitor food quality, since plant uptake is one of the main pathways through which potentially toxic elements (PTE) enter the food chain (Fernández-Caliani et al. 2019). Gupta et al. (2018) reviewed the several factors that affect the rate of trace element accumulation, toxicity mechanism, and effects on vegetables and humans and described various toxicity indices for health risk assessment. Urban soils are characterized by a great variability in their physicochemical parameters and properties due to the high anthropization effects, the great variety and combination of sources of disturbance, the diversity of cultivation practices, and the ample range of contamination levels (Bidar et al. 2020). Vegetables and soils can be contaminated by different PTEs. Among them, As, Cd, Co, Cr, Ni, and Pb can be extremely toxic for living organisms.

Different authors reported high levels of different PTEs in urban garden soils from different cities, exceeding the recommended limits for agricultural use (Pruvot et al. 2006; Peris et al. 2007; Ramos and Pinto 2008; Kabala et al. 2009; Izquierdo et al. 2015; De Miguel et al. 2017; López et. al. 2019; Taylor et al. 2021). Cetin et al. (2022) found that Ni and Co soil concentrations increased with high levels of human activities and recommend not to use the plants grown in these soils for food purposes. Very recently, it was highlighted the prevalence of harmful concentrations of contaminants in organically managed urban community gardens near Seattle, Washington (Malone 2022), and Romero-Baena et al. (2021) reported high concentrations of As, Cu, Pb, and Zn in soils from family gardens of Riotinto mining area.

Lead contamination has been appearing as the most common risk factor in urban orchards because of its high level in soils and because of its ubiquity as an environmental pollutant (Mielke et al. 1983; Pelfrêne et al. 2019; Paltseva et al. 2022). The presence and effects of PTEs are often not taken into account when establishing orchards (Malone 2022). Air pollution also contributes to trace metal contamination of vegetables grown in high-traffic areas of developed countries (Mok et al. 2014). The concentration of some PTE levels was studied in vegetables cultivated in urban garden from different cities of the world (Kohrman and Chamberlain 2014; Arrobas et al. 2016; Antoine et al. 2017; França et al. 2017; Pelfrêne et al. 2019; Parveen et al. 2020; Hiller et al 2022), with different results about the safety of consuming these vegetables, but only a few PTEs were studied. Moreover, data about the safety of vegetables cultivated in gardens located in different sites of Mediterranean areas, including mining areas, are scarce (López et al 2019; Rossini-Oliva et al. 2020; Rossini-Oliva and López 2021).

Paradoxically, it has been found that soil contamination by PTEs is not usually identified among urban horticulturists as a common hazard in urban agriculture, and soil and crop controls are often not carried out in urban gardens (Hunter et al. 2019). Nevertheless, a common view among urban agriculture researchers and policymakers is that on-site field studies in urban areas are still rather scarce but will be crucial for determining the health risks of urban horticulture (Ganguli et al. 2024). Urban agriculture has many benefits (Gliessman 2017), and urban food production produces a reduction of food transportation, packaging, and energy use contributing to the climate change mitigation (Cleveland et al 2017), but consumers need to be sure to eat safe food. In a very recent review by Ganguli et al. (2024), it is noted that the scope of research into the risks of urban agriculture remains relatively limited focusing mainly on the social benefits.

This study aimed (1) to determine whether consumption of the urban garden produce represents a potential human health risk based on comparison with guidance values; (2) to compare the concentrations of PTEs in the most frequently consumed vegetables (leafy, bulbous, and fruiting species) from different urban gardens located in three cities of Andalusia (Seville, Cordoba, and Huelva), with one mining area and a peri-urban area; (3) to study soil-to-vegetable PTE transfer; and (4) to compare PTE concentrations in urban garden produce with concentrations determined in local groceries.

Thus, the objectives of this study are to evaluate a high number of PTEs in soils and in a wide range of vegetables cultivated in urban gardens in order to evaluate possible risks for human health consumption in areas with a Mediterranean climate, which until now, according to the authors’ knowledge, has been very scarce considered.

## Materials and methods

### Plant and soil sampling

Vegetable and soil samples were taken from urban gardens in the towns of Nerva (5127 inhabitants), Minas de Riotinto (3778 inhabitants), and Alcala de Guadaira (75,256 inhabitants); in the cities of Huelva (144,258 inhabitants), Córdoba (325,708 inhabitants), and Seville (688,711 inhabitants); and in peri-urban gardens of Utrera (51,402) municipalities, all in southwestern Spain. The towns Minas de Riotinto and Nerva are located in the Iberian Pyrite Belt mining region, one of the heaviest metal-contaminated fluvial–estuarine systems in the world (Amils et al. 2007). Mining has been carried out in this area since ancient times, and the sampling sites were very close to operating mines. The cities of Utrera, Alcala de Guadaira, Huelva, Cordoba, and Seville differ mainly in their population, with Alcalá de Guadaira and Huelva having the largest industrial hubs. The list of urban gardens, their locations, and any relevant information about the surroundings are shown in Table 1 Supplementary Material.

Vegetable samples (lettuce, chard, tomato, onion, pepper, zucchini, and eggplant) together with composite soil samples from the adjacent area to vegetable roots (0–20 cm depth) were collected in the different urban gardens during 2021–2023. Most of the plots sampled were in social gardens with individual plots of about 100 m^2^ in which several vegetables were grown simultaneously during each season. Thus, the number of samples collected from a particular plot/garden varied according to vegetable availability. In each plot, soil samples were joined forming a composite sample, if they corresponded to different vegetables. As they are small individual plots, management (modifications, irrigation, tillage) is carried out jointly for the entire plot. In all the orchards, cultivation is carried out under organic farming practices.

The samples from the peri-urban garden of Utrera, located more than 23 km away from Seville city and in a small city with no industrial activities, low traffic density, and a lower human interference, were considered a priori as probably little exposed to any type of anthropic contamination. In addition, the same vegetables were purchased in a local market of Seville to compare with city orchards.

A total of 282 plant samples and 102 soil samples were analyzed. During March 2022, an important haze episode occurred in Seville, due to Saharan dust transport, so lettuce and chard were sampled after a “red rain” event in three urban gardens of the city to study the consequences for plant element composition. These data were analyzed separately.

### Plant and soil analysis

In the lab, plant samples were previously washed with tap water and then with distilled water. Fruits and roots were peeled and cut into small pieces. Samples were oven-dried at 70 °C, weighed, and finely ground in a plant mill. A portion of 0.25 g of each sample was digested in a digestion block, with a nitric acid solution (Rossini-Oliva and López-Núñez 2021). After digestion, they were diluted to a final volume of 50 mL with deionized water. PTE contents (As, B, Ba, Cd, Co, Cr, Cu, Mo, Ni, Pb, and Zn) were determined in this solution by inductively coupled plasma mass spectrometer (ICP-MS). The accuracy of the analysis was verified by analyzing a standard reference material (Apple Leaves NIST 1515).

Soil samples were dried at 100 °C, ground, and sieved with a 0.5-mm size sieve. Soil pH and conductivity were determined in 1:2.5 and 1:5 water extracts respectively. Total elemental contents of PTEs in soil samples were determined by using portable X-ray fluorescence (pXRF) (analyzer Niton XL3t 950s GOLDD + XRF, Thermo Scientific Inc., Billerica, MA, USA). In-built soil analysis mode and a scan time of 90 s were used. The finely ground samples were measured in an XRF container (model SC-4331, 26 mm internal diameter, 24 mm height, Premier Lab Supply Inc., Port St. Lucie, FL, USA) capped with a 4-µm propylene film (model 240,255, 63 mm diameter, Premier Lab Supply Inc., Port St. Lucie, FL, USA). The container was placed in the window of the analyzer and scanned three times, and the average values from three replicate scans were selected. The reference material SdAR-M2 (International Association of Geoanalysts 2015) was used to assess the accuracy and stability of the pXRF instrument. It is assumed a maximum 20% relative difference of the true value is acceptable for this technique (USEPA 2007).

### Environmental risk

For the evaluation and interpretation of the concentration results in soils, the regulatory regional reference values were considered. These reference values (As, 36 mg kg^−1^; Cu, 595 mg kg^−1^; Cr(III), 10,000 mg kg^−1^; Ni, 1540 mg kg^−1^; Pb, 275 mg kg^−1^; Zn, 10,000 mg kg^−1^) are the threshold values (TV) for agricultural use indicating the need for further assessment. The PTE measured concentrations in the garden soils (C^i^) were also compared with the background concentrations (C^i^_bk_) for the specific area given by Aguilar-Ruíz et al. (1998). For Nerva and Riotinto sites, background values corresponding to the South Portuguese area were selected. This area is part of the Iberian Pyrite Belt, one of the world’s largest reserves of massive sulfides, which ranges from the center of Andalusia to the Portuguese region of Aljustrel. For the rest of the locations, the background values of the Guadalquivir river basin have been used. The Guadalquivir basin is filled with Neogene materials (Miocene-Pliocene), and there are also Quaternary deposits, mostly belonging to the terraces and alluvials of the great rivers and their tributaries.

Additionally, the following contamination indexes have been used:

The concentration factors (CF^i^) of the metals/metalloids As, Cr, Cu, Ni, Pb, and Zn, defined as the ratio between the concentration of the metal in each soil and the background values for the region, were calculated as

$${{\text{CF}}}^{{\text{i}}} = {{{\text{C}}}^{{\text{i}}}}_{{\text{bk}}} / {{\text{C}}}^{{\text{i}}}$$

CF^i^ accounts for the contamination of single elements. For this risk index approach, CF^i^ < 1 is indicative of low contamination, 1 < CF^i^ < 3 is indicative of moderate contamination, 3 < CF^i^ < 6 is indicative of considerable contamination, and CF^i^ > 6 of very high contamination (Hakanson 1980).

To account for overall contamination by all PTEs, we use the pollution load index (PLI). Although originally this index was defined in sediments, it is also used for soils (Madejón et al. 2017). PLI is calculated as the *n*th root of the product of the obtained CF^i^. Values of PLI close to 1 indicate no PTE contamination, while values above 1 indicate soil contamination.

In the soil analyses, some samples (14 in the case of As, 37 for Cr, 5 for Cu, and 15 for Ni) gave results below the detection limit (LOD) of the technique. To avoid bias, values below the LOD were replaced by the lowest value of all those obtained.

### Human health risk assessment

The hazard quotient (HQ) and hazard index (HI) were used to establish non-carcinogenic risks (USEPA 2010). HQ is the ratio between exposure to the potentially toxic elements and the standard reference oral dose (RfD). RfD is the highest level at which no adverse health effects are expected. If the ratio is lower than one, there will be no obvious risk. However, if the HQ is > 1, then there is a possibility that adverse health effects could be experienced. The formula used for calculating HQ is the following (United States Environmental Protection Agency methodology):

$$\mathrm{HQ}=\frac{{\text{EDI}}}{{\text{RfD}}}$$

EDI is the estimated daily intake that can be calculated as:

$$EDI=\frac{Cm\times Ir\times Ef\times Ed}{BW\times AT}$$

where Cm is the concentration of element in the vegetable (mg kg^−1^ dry weight); IR is the daily vegetable ingestion rate (kg per day); Ef is the exposure frequency (day/years); Ed is the exposure duration (years); BW is the average body weight (70 kg for adult a); RfD is the oral reference dose for the metal (mg kg^−1^ of body weight per day); and AT is the average exposure time for non-carcinogenic effects (30 × 365 days/year). Since the ingestion rate was in fresh weight, EDI was multiplied by 0.085 (Junta de Andalucia 2017). RfD values are 0.0003, 0.2, 0.2, 0.001, 0.0003, 0.003, 0.04, 0.02, 0.0035, and 0.3 mg/kg/day for As, B, Ba, Cd, Co, Cr, Cu, Ni, Pb, and Zn, respectively (EPA 2008; USEPA 2010, 2015; WHO 1993). Ed was established as 30 years for adults. IR was estimated according to the Regional Annual Consumption data (Ministerio de Agricultura, Pesca y Alimentación 2022).

Since the health effect is due to more than one element, the hazard index (HI) for each studied vegetable was calculated as the total hazard quotient (USEPA 2010):

$$\mathrm{HI }={{\text{TQ}}}_{{\text{As}}} + {{\text{TQ}}}_{{\text{B}}} + {{\text{TQ}}}_{{\text{Ba}}} +{{\text{TQ}}}_{{\text{Cd}}} + {{\text{TQ}}}_{{\text{Co}}}+ {{\text{TQ}}}_{{\text{Cr}}}+ {{\text{TQ}}}_{{\text{Cu}}}+ {{\text{TQ}}}_{{\text{Mo}}}+ {{\text{TQ}}}_{{\text{Ni}}}+ {{\text{TQ}}}_{{\text{Pb}}}+ {{\text{TQ}}}_{{\text{Zn}}}$$

If the TQ or HI is > 1, there is the potential for adverse non-carcinogenic health effects (USEPA 2010).

### Carcinogenic risk (CR) of As

Carcinogenic risk is the health risk from carcinogens. The target carcinogenic risk (CR) can be calculated as (USEPA 1989):

$$CR=\frac{Cm\times Ir\times Ef\times Ed\times SF}{BW\times AT}$$

AT in this case is 78 × 365 days (28,470 days) since it is calculated for exposure duration over the lifetime (Junta de Andalucía 2017); SF is the oral slope factor (mg/kg/day)^−1^. The oral slope factor from the Integrated Risk Information System (USEPA 2010, 2015) is 1.5 for As. Values of CR higher than 10^−5^ are considered unacceptable (RD/9/2005).

### Statistical analysis

Data were first checked for normal distribution by Shapiro–Wilk’s test. If data were normally distributed, differences in PTE levels in each species or soil from different urban gardens were compared by ANOVA and post hoc Tukey test. Non-parametric Kruskal–Wallis test was used when data were not normally distributed followed by Mann–Whitney *U* test. Data of vegetables were separated among fruiting, bulbous, and leafy species to test differences in PTE accumulation between groups. The translocation factor (TF) was calculated as the ratio of the element concentration in the edible parts of the vegetables and the total element concentration in the soil where the plant had grown. This factor is useful to estimate the capacity of the species to accumulate PTEs in the edible part. The soil-to-plant transfer factors (TF) were calculated as the ratio of the element concentration in the edible parts of the vegetables and the total element concentration in the soil where the plant had grown. This factor is useful to estimate the capacity of the species to accumulate PTEs in the edible part.

## Results

### Soils

Table 1 shows the descriptive statistics for PTE concentrations and pH values in each urban garden soil, including the average, maximum, and minimum concentration values. To facilitate the comparison of the data, they have been grouped in Table 2 according to the type of orchard in peri-urban (UTR site), mining (NER and TIN sites), and city (cities).

**Table 1:** Concentrations of PTEs (mean, minimum and maximum values) and values of pH in the soils of the different urban gardens

Garden	*n* ^1^	pH			As			Cr			Cu		
		Mean	Min	Max	Mean	Min	Max	Mean	Min	Max	Mean	Min	Max
UTR	3	7.85	7.84	7.86	5.5	4.6	6.1	22.2	< LD	25.9	29.0	23.8	36.9
LUC	3	7.44	7.37	7.49	8.2	8.2	8.2	20.4	< LD	20.4	17.9	17.8	18.0
ALA	15	7.75	7.39	8.02	13.8	9.8	18.3	30.0	20.4	61.5	45.1	34.4	61.0
ALC	13	7.67	7.49	7.98	7.2	< LD	11.0	20.8	< LD	26.0	93.8	28.0	265.0
GUA	18	7.93	7.57	8.41	9.1	< LD	19.7	32.7	< LD	52.5	28.3	17.8	46.4
ELE	2	8.38	8.13	8.63	9.1	9.1	9.1	29.9	28.3	31.5	21.8	17.8	25.8
HER	18	7.74	7.28	8.11	8.6	< LD	12.5	39.9	< LD	58.1	38.5	20.0	86.7
MI2	5	7.73	7.66	7.87	12.4	10.5	14.8	39.9	32.6	46.0	36.2	28.8	59.3
TRI	6	7.85	7.09	8.28	6.4	4.6	7.9	21.5	21.5	21.5	19.1	17.8	22.1
TOR	3	8.66	8.19	8.90	8.4	7.8	9.8	22.9	< LD	27.8	23.0	17.8	27.5
ASO	4	7.53	7.50	7.60	14.1	10.2	19.1	36.7	< LD	58.7	55.0	44.1	70.5
LEV	3	7.56	7.07	8.00	14.8	13.1	17.5	45.6	29.5	62.1	58.7	44.2	87.3
MOR	3	6.97	6.50	7.56	9.3	8.4	10.6	33.7	< LD	51.4	69.4	44.5	82.6
NER	2	6.51	5.96	6.81	87.2	85.5	88.9	90.5	64.0	117.0	333.1	268.0	398.2
TIN	5	7.25	6.50	7.75	167.8	48.9	608.9	110.2	53.0	139.2	155.8	128.5	232.6
					Ni			Pb			Zn		
UTR					30.5	27.8	32.8	14.3	13.1	16.3	36.0	20.4	52.2
LUC					28.0	< LD	29.1	17.2	16.4	18.2	36.7	27.0	55.3
ALA					53.9	37.5	68.1	55.3	39.7	67.1	94.5	77.2	127.1
ALC					31.9	< LD	41.0	18.2	14.0	24.0	112.3	45.0	247.0
GUA					39.0	< LD	50.2	29.4	16.0	64.5	54.1	36.7	107.7
ELE					43.2	< LD	58.9	27.4	26.4	28.3	67.8	53.2	82.5
HER					37.0	27.5	56.2	18.5	13.4	23.0	80.8	59.1	98.8
MI2					53.0	35.3	63.1	55.3	43.1	72.0	69.8	49.8	97.3
TRI					42.6	30.4	53.8	17.1	14.6	23.6	43.8	35.1	59.3
TOR					56.2	49.2	61.0	27.6	26.6	28.3	52.6	51.5	53.4
ASO					48.9	43.4	55.7	54.0	23.1	92.9	90.5	79.2	105.8
LEV					52.4	45.4	64.6	57.5	48.5	64.8	74.6	59.9	98.0
MOR					35.2	< LD	46.8	33.0	20.2	43.4	95.2	56.0	132.2
NER					40.7	< LD	54.0	359.6	283.9	435.4	458.5	433.0	484.0
TIN					41.3	< LD	55.0	346.4	144.2	1079.6	249.7	207.1	373.0

^1^ *n*, number of samples; < LD, below detection limit

**Table 2:** Statistics for the concentration of PTEs (mean, minimum, and maximum values in mg kg^−1^) and pH of the soils of the different types of urban gardens

		Peri-urban	City	Mining
		3	93	7
pH	Median	7.85	7.77	6.81
	Mean	7.85	7.74	6.97
	Minimum	7.84	6.50	5.96
	Maximum	7.86	8.90	7.74
As	Median	5.8	9.0	65.9
	Mean	5.5^a^	9.9^b^	144.7^c^
	Minimum	4.6	< LD	48.9
	Maximum	6.1	19.7	608.9
Cr	Median	20.4	27.8	117.0
	Mean	22.2^a^	31.5^a^	104.5^b^
	Minimum	< LD	< LD	53.0
	Maximum	25.9	62.1	139.2
Cu	Median	26.5	36.2	146.2
	Mean	29.0^a^	44.8^a^	206.5^b^
	Minimum	23.8	17.8	128.5
	Maximum	36.9	265.0	398.2
Ni	Median	30.9	40.9	41.6
	Mean	30.5^a^	42.0^a^	41.1^a^
	Minimum	27.8	< LD	< LD
	Maximum	32.8	68.1	55.0
Pb	Median	13.5	22.9	189.8
	Mean	14.3^a^	32.1^b^	350.2^c^
	Minimum	13.1	13.4	144.2
	Maximum	16.3	92.9	1079.6
Zn	Median	35.3	75.3	228.1
	Mean	36.0^a^	77.4^b^	309.3^c^
	Minimum	20.4	27.0	207.1
	Maximum	52.2	247.0	484.0

< *LD*, below detection limit; different letters among garden types indicate significant differences following the Kruskal–Wallis non-parametric test

In general, the soil pH was neutral to moderately alkaline, with values between 6.5 and 8.9 in all cases. In the mining area, soils showed pH values close to neutrality. In urban city soils, the mean value of pH was always greater than 7 with two orchards showing values greater than 8.

The average concentration of As was 5.5 mg kg^−1^ in the peri-urban garden, 9.9 mg kg^−1^ in the urban city gardens, and much greater, 145 mg kg^−1^ in the mining gardens, reaching a maximum value of 609 mg kg^−1^ specifically in the TIN garden. Arsenic concentrations in the three sites were statistically different. The average concentration of Cr was 22.2 mg kg^−1^ in the peri-urban gardens and similar (31.5 mg kg^−1^) in the urban city orchards but significantly higher (104 mg kg^−1^) in the mining gardens. The average concentration of Cu in the peri-urban garden (UTR) was similar to that of urban city gardens but statistically lower than in the mining urban gardens (206 mg kg^−1^). An ample variation in Cu concentration was observed in both urban and mining gardens: 18–265 mg kg^−1^ and 128–398 mg kg^−1^ respectively. The average concentration of Ni was similar in all sites reaching a maximum value of 68 mg kg^−1^ in one urban plot (Alamillo Park). The average Pb concentration (14.3 mg kg^−1^) was lower in the peri-urban garden than in other areas, while the highest concentration (350 mg kg^−1^) was found in the mining orchards reaching up to 1080 mg kg^−1^ in the TIN garden. The mean Zn concentration was the lowest in peri-urban gardens (36 mg kg^−1^), while it was more than doubled in city areas, whereas the highest values (309 mg kg^−1^) were observed in the mining area.

**Fig. 1:**
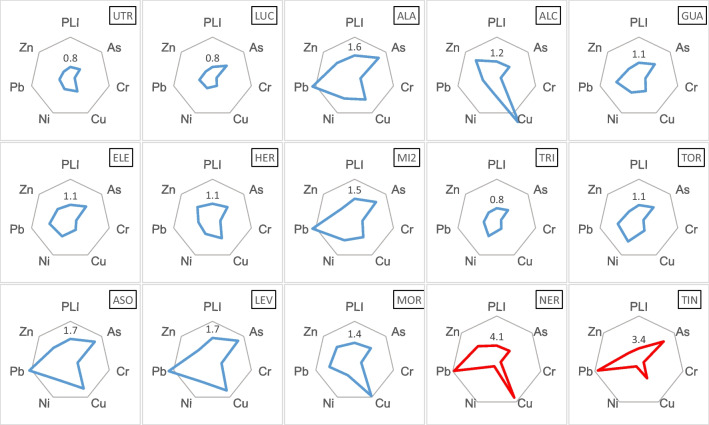
Average concentration factors (CF) for the studied PTEs and pollution load index (PLI) in each garden. For the drawings in blue, the scale indicated by the hexagons corresponds to 3 units, while for the gardens in red, the scale corresponds to 12 units. Figures indicate the PLI value

The CF and PLI means for PTE in each garden are shown in Fig. 1. The CF was close to 1 or lower than 1 in the peri-urban orchard (UTR) as well as in some city orchards: LUC, GUA, and TRI. For Cr, the CF remained in values around the unit in all of the gardens. But in several city orchards (ALA, MI2, ASO, LEV, MOR), the CF of As, Cu, and Pb had values close to 3 (peaks of the stars of Fig. 1). In mining orchards, the same PTEs reached CF in the order of 6–12 (red lines). The lowest PLI, 0.8, was found in the peri-urban garden and the TRI and LUC urban city garden. In the city orchards, PLI values ranged from 0.8 to 1.7 with the highest numbers corresponding to ASO and LEV, sites located in Córdoba city. The highest PLI values were found in gardens from the mining area (3.4 and 4.1).

### Plant

The mean concentration and standard deviations of PTEs in the vegetables from different urban garden areas are shown in Table 3. Significant differences in PTEs were observed in vegetables from urban gardens of the three areas tested (peri-urban, mining, and city) and the local market, varying according to the element and species. For onion, As mining > all others, city > peri-urban; market > peri-urban, city = market; B mining > peri-urban, market > all areas, city = peri-urban; Cu mining > city, city = peri-urban, mining = peri-urban, market > all areas; Mo mining > all areas, market < all areas, city = peri-urban; Pb mining > city > market = peri-urban, city > market, peri-urban > market, city = peri-urban; Zn content was only statistically higher in plants from market > city. For pepper, As mining > all areas, city > peri-urban = market; Co peri-urban > mining > city = market, city = market, city > mining, city = peri-urban; Pb city = peri-urban = market, mining < peri-urban < city; mining = market, Zn city = peri-urban, peri-urban > mining > market, city = market. For tomato, B, Cu, and Zn were only higher in mining area with respect to city and market, city = market, city = peri-urban; As mining > all areas, city < peri-urban; Cd peri-urban > all areas, city = market = mining; Co city = peri-urban = market, peri-urban > mining; mining < city < market; Cr peri-urban < mining, city = market; city = peri-urban, city > mining, market > mining. For eggplants, As mining > peri-urban > city, mining = market, market = city and city > peri-urban; Cd peri-urban > all areas; city = market; Co mining < city < peri-urban < market, market = peri-urban and city, city = peri-urban; Mo peri-urban > mining = market and city; market > city > mining; Pb peri-urban > mining = city > market; market = city, city > mining; Zn peri-urban > mining > city, peri-urban = market; city = market; mining = city and market. For lettuce As market < peri-urban < city, city = peri-urban; B peri-urban > market, city = market, city > peri-urban; Ba city = peri-urban, city > market; peri-urban > market; Cd peri-urban > the city > market; city > market; Cr peri-urban = city; city > market, peri-urban = market; Cu peri-urban > city > market; market < city < peri-urban, city = market; Mo city > peri-urban > market; Ni city > peri-urban, city = market, peri-urban < city < market; Pb city = peri-urban, market < city < peri-urban. For chard, As mining > all other areas, city > market and peri-urban; Ba peri-urban > all other areas, city = market; Cd mining > all other areas, city = peri-urban, city = market; Co market > all the other areas, city = peri-urban; Pb mining > city > market, city = peri-urban, city > market, peri-urban > market. For zucchini, As in mining > city > peri-urban; market = all areas, city > peri-urban; Cu mining > peri-urban, city = market = mining = peri-urban; Pb market = peri-urban, peri-urban = city, also peri-urban = mining, mining > city; city = market; Zn peri-urban > market > city, market = city; mining > city and market.

**Table 3:** Mean values (mg/kg) ± standard deviation of elements in vegetables from market (Mk) and cultivated in mining (M), urban (U), and peri-urban gardens (P)

Sample	As	B	Ba	Cd	Co	Cr	Cu	Mo	Ni	Pb	Zn
Onion (P)	0.031 ± 0.002	14.92 ± 0.145	3.476 ± 0.050	0.044 ± 0.015	0.088 ± 0.032	0.106 ± 0.022	6.124 ± 0.187	0.225 ± 0.040	0.297 ± 0.155	0.249 ± 0.157	37.33 ± 2.745
Onion (M)	0.558 ± 0.331	16.79 ± 0.895	5.153 ± 1.779	0.052 ± 0.015	0.087 ± 0.017	0.346 ± 0.094	8.199 ± 1.382	1.960 ± 0.837	0.333 ± 0.024	0.300 ± 0.129	27.39 ± 4.751
Onion (U)	0.058 ± 0.030	11.75 ± 6.689	9.265 ± 8.242	0.093 ± 0.109	0.092 ± 0.073	0.518 ± 0.680	6.461 ± 5.346	0.883 ± 1.097	0.700 ± 0.825	0.153 ± 0.132	23.75 ± 11.33
Onion (Mk)	0.043 ± 0.000	22.90 ± 0.557	5.667 ± 0.029	0.068 ± 0.006	0.121 ± 0003	0.199 ± 0.064	12.36 ± 0.125	0.104 ± 0.001	0.677 ± 0.015	0.034 ± 0.006	40.70 ± 1.323
Significant	*	*	ns	ns	Ns	ns	*	*	ns	*	*
Pepper (P)	0.014 ± 0.003	12.32 ± 0.249	1.037 ± 0.014	0.235 ± 0.082	0.295 ± 0.121	0.140 ± 0.005	12.79 ± 0.619	0.146 ± 0.001	0.331 ± 0.048	0.229 ± 0.147	31.52 ± 0.790
Pepper (M)	0.062 ± 0.030	12.65 ± 2.075	0.710 ± 0.551	0.069 ± 0.023	0.050 ± 0.045	0.180 ± 0.141	8.41 ± 3.314	0.291 ± 0.149	0.387 ± 0.374	0.055 ± 0.029	17.13 ± 4.047
Pepper (U)	0.036 ± 0.019	13.36 ± 5.232	1.041 ± 1.020	0.114 ± 0.059	0.166 ± 0.079	0.337 ± 0.566	9.64 ± 3.201	0.445 ± 0.396	0.479 ± 0.426	0.176 ± 0.119	22.06 ± 6.568
Pepper (Mk)	0.031 ± 0.028	9.450 ± 3.748	0.370 ± 0.153	0.086 ± 0.078	0.119 ± 0.089	0.302 ± 0.204	5.76 ± 2.207	0.846 ± 0.341	0.391 ± 0.170	0.104 ± 0.090	16.90 ± 6.222
Significant	*	ns	ns	ns	*	ns	ns	ns	ns	*	*
Tomato (P)	0.017 ± 0.002	11.31 ± 0.182	0.990 ± 0.028	0.228 ± 0.017	0.187 ± 0.035	0.102 ± 0.021	9.352 ± 0.859	0.796 ± 0.049	0.160 ± 0.008	0.166 ± 0.026	28.72 ± 1.538
Tomato (M)	0.077 ± 0.055	14.29 ± 2.492	0.772 ± 0.238	0.103 ± 0.018	0.039 ± 0.032	0.227 ± 0.053	10.86 ± 1.169	0.807 ± 0.241	0.274 ± 0.120	0.089 ± 0.025	33.17 ± 5.172
Tomato (U)	0.007 ± 0.005	10.92 ± 3.343	0.913 ± 0.551	0.095 ± 0.054	0.136 ± 0.107	0.237 ± 0.396	7.810 ± 2.641	0.858 ± 0.509	0.220 ± 0.189	0.165 ± 0.167	20.10 ± 6.027
Tomato (Mk)	< dl	7.450 ± 4.172	1.585 ± 1.213	0.073 ± 0.009	0.198 ± 0.110	0.292 ± 0.008	4.337 ± 2.351	0.379 ± 0.233	0.460 ± 0.134	0.056 ± 0.015	12.24 ± 7.551
Significant	*	*	ns	*	*	*	*	ns	ns	ns	*
Eggplant (P)	0.011 ± 0.002	21.64 ± 0.885	1.386 ± 0.412	0.259 ± 0.009	0.114 ± 0.014	0.103 ± 0.079	13.55 ± 5.638	0.815 ± 0.025	0.175 ± 0.089	0.135 ± 0.018	38.50 ± 3.240
Eggplant (M)	0.181 ± 0.125	15.33 ± 3.153	0.984 ± 0.647	0.097 ± 0.028	0.018 ± 0.015	0.163 ± 0.091	8.407 ± 1.76	0.405 ± 0.112	0.276 ± 0.107	0.061 ± 0.015	20.87 ± 4.712
Eggplant (U)	0.109 ± 0.086	17.19 ± 3.896	1.373 ± 1.755	0.148 ± 0.070	0.117 ± 0.056	0.192 ± 0.329	8.495 ± 2.67	0.632 ± 0.461	0.190 ± 0.069	0.213 ± 0.112	22.01 ± 4.931
Eggplant (Mk)	0.250 ± 0.059	18.85 ± 0.212	0.806 ± 0.0229	0.060 ± 0.007	0.093 ± 0.005	0.193 ± 0.015	10.65 ± 0.495	2.098 ± 0.205	0.222 ± 0.003	0.090 ± 0.005	22.95 ± 1.485
Significant	*	ns	ns	*	*	ns	ns	*	ns	*	*
Lettuce (P)	0.094 ± 0.009	43.17 ± 0.273	6.041 ± 0.366	1.140 ± 0.055	0.102 ± 0.004	0.294 ± 0.018	11.74 ± 0.207	0.207 ± 0.006	0.263 ± 0.013	0.420 ± 0.140	75.64 ± 2.516
Lettuce (U)	0.164 ± 0.081	25.32 ± 12.09	11.10 ± 9.368	0.291 ± 0.329	0.163 ± 0.119	0.634 ± 0.614	8.853 ± 2.537	0.936 ± 0.993	0.490 ± 0.390	0.323 ± 0.186	43.13 ± 23.39
Lettuce (Mk)	0.037 ± 0.005	40.08 ± 1.164	2.205 ± 0.229	0.128 ± 0.008	0.111 ± 0.044	0.300 ± 0.080	6.995 ± 0.292	0.291 ± 0.036	0.508 ± 0094	0.067 ± 0.019	38.53 ± 4.870
Significant	*	*	*	*	Ns	*	*	*	*	*	ns
Chard (P)	0.027 ± 0.002	34.16 ± 1.405	81.96 ± 8.969	0.123 ± 0.025	0.114 ± 0.037	0.188 ± 0.056	9.072 ± 0.649	0.401 ± 0.081	0.204 ± 0.057	0.278 ± 0.078	38.95 ± 1.623
Chard (M)	0.272 ± 0.242	39.24 ± 7.196	21.26 ± 10.51	0.290 ± 0.111	0.118 ± 0.025	0.407 ± 0.227	10.95 ± 2.228	1.132 ± 0.414	0.600 ± 0.293	0.698 ± 0.535	53.30 ± 35.06
Chard (U)	0.069 ± 0.044	29.49 ± 13.25	29.76 ± 21.79	0.142 ± 0.089	0.192 ± 0.082	0.481 ± 0.457	10.31 ± 7.456	1.115 ± 1.199	0.455 ± 0.381	0.217 ± 0.127	34.13 ± 8.405
Chard (Mk)	0.021 ± 0.012	27.65 ± 1.449	25.22 ± 6548	0.094 ± 0.045	0.274 ± 0.013	0.284 ± 0.041	7.987 ± 1.530	0.620 ± 0.045	0.348 ± 0.099	0.118 ± 0.026	42.10 ± 4.399
Significant	*	ns	*	*	*	ns	ns	ns	ns	*	ns
Zucchini (P)	0.012 ± 0.000	32.18 ± 0.587	3.715 ± 0.206	0.055 ± 0.012	0.126 ± 0.023	0.129 ± 0.043	12.92 ± 0.11	2.346 ± 0.097	0.232 ± 0.028	0.232 ± 0.052	87.15 ± 4.311
Zucchini (M)	0.336 ± 0.270	29.72 ± 6.173	2.281 ± 1.47	0.054 ± 0.008	0.108 ± 0.011	0.292 ± 0.081	17.90 ± 4.91	2.219 ± 0.472	0.469 ± 0.277	0.522 ± 0.442	74.00 ± 4.690
Zucchini (U)	0.029 ± 0.019	20.47 ± 8.509	3.851 ± 2.473	0.050 ± 0.036	0.192 ± 0.144	0.592 ± 0.742	11.76 ± 4.51	1.794 ± 1.091	1.531 ± 1.612	0.111 ± 0.082	48.28 ± 19.08
Zucchini (Mk)	0.045 ± 0.007	23.10 ± 5.374	1.826 ± 0.146	0.051 ± 0.016	0.140 ± 0.028	1.385 ± 0.701	7.83 ± 0.728	1.393 ± 0.194	0.704 ± 0.207	0.096 ± 0.009	29.90 ± 1.980
Significant	*	ns	ns	ns	ns	ns	*	ns	ns	*	*

**Fig. 2:**
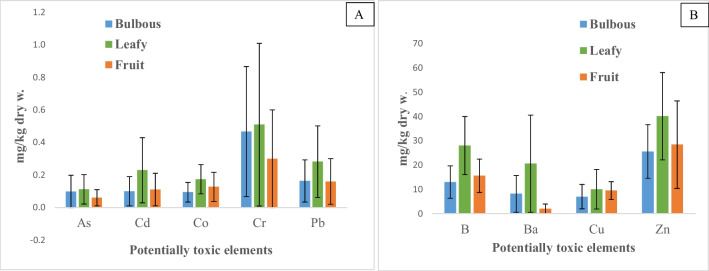
**A**, **B** Mean ± standard deviation of concentration of potentially toxic elements in the different plant species groups

Leafy vegetables were generally able to accumulate more PTEs (Fig. 2A, B) than bulbous and fruiting species, and statistical differences were not observed for Mo and Ni in the three groups of vegetables (data not shown). For As and Cr, leafy and bulbous species had similar accumulation capacity. Chard was the species with the highest Ba accumulation, and B was particularly accumulated by chard and lettuce. Lettuce had also the highest capacity to accumulate Cd while Pb was particularly accumulated by lettuce and chard.

**Fig. 3:**
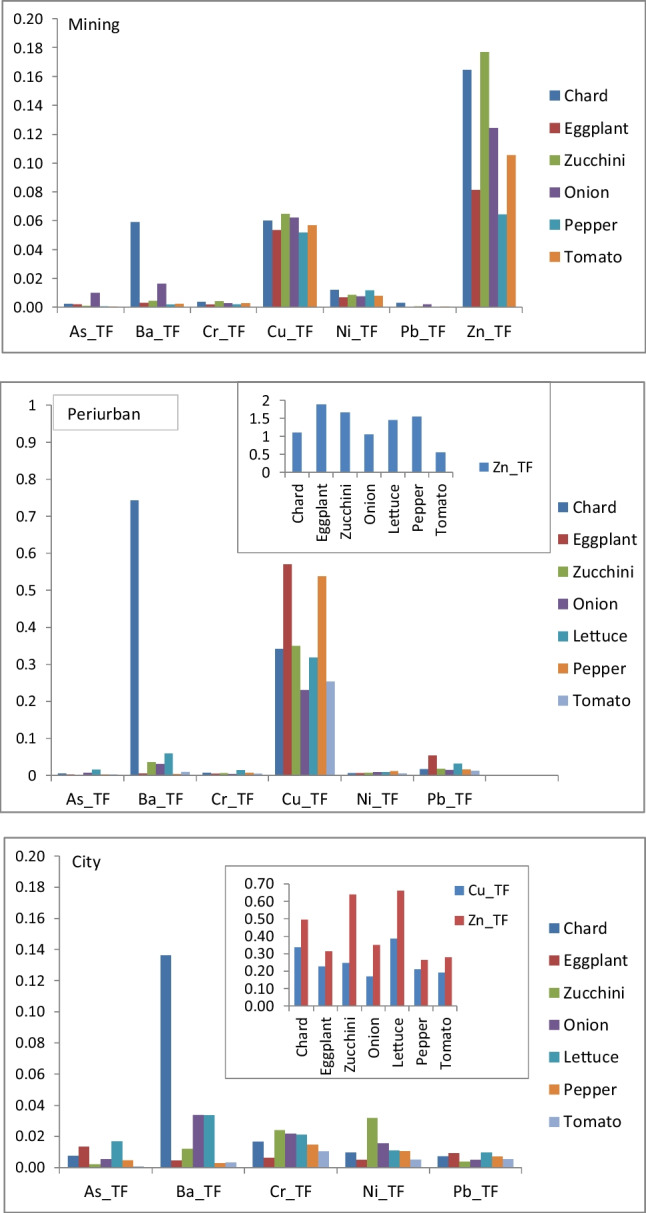
Soil-to-plant transfer factors (TF) of PTEs in the studied vegetables cultivated in the different urban gardens

Values of transfer factors (TF) are shown in Fig. 3. Generally, all potentially toxic elements (As, Ba, Cr, Cu, Ni, Pb, and Zn) had TF values below 1 for all studied species, and differences varied from element to element. For Ba, chard > than in other species; for Zn, lettuce showed a similar value to zucchini, and both had greater TF than the rest. For Cu, chard = lettuce > others; for Pb, chard = lettuce = eggplant > others; for Cr and Ni depended on the areas; for As, lettuce > others and in mining area onion > others.

In Seville, haze episodes (“*calima*”) occur with a certain frequency in particular weather conditions. Haze carries dust and sand particles from Africa and plants may absorb them. Dust is a source of plant nutrients, such as Fe, but can also transport PTE (Menendez et al. 2007). During these episodes, air quality is extremely unfavorable for humans, according to the Spanish Ministry for Ecological Transition (Miteco 2023). Figure 1 (Supplementary material) shows differences in PTE observed for lettuce and chard during periods of haze and no haze. The Cu content in lettuce sampled after haze was higher compared with the un-haze samples, and for chard, an enrichment of As, B, Co, Cr, Ni, and Fe (we have no data of Fe for lettuce) was observed (Fig. 1 Supplementary Material).

### Health risk assessment

**Fig. 4:**
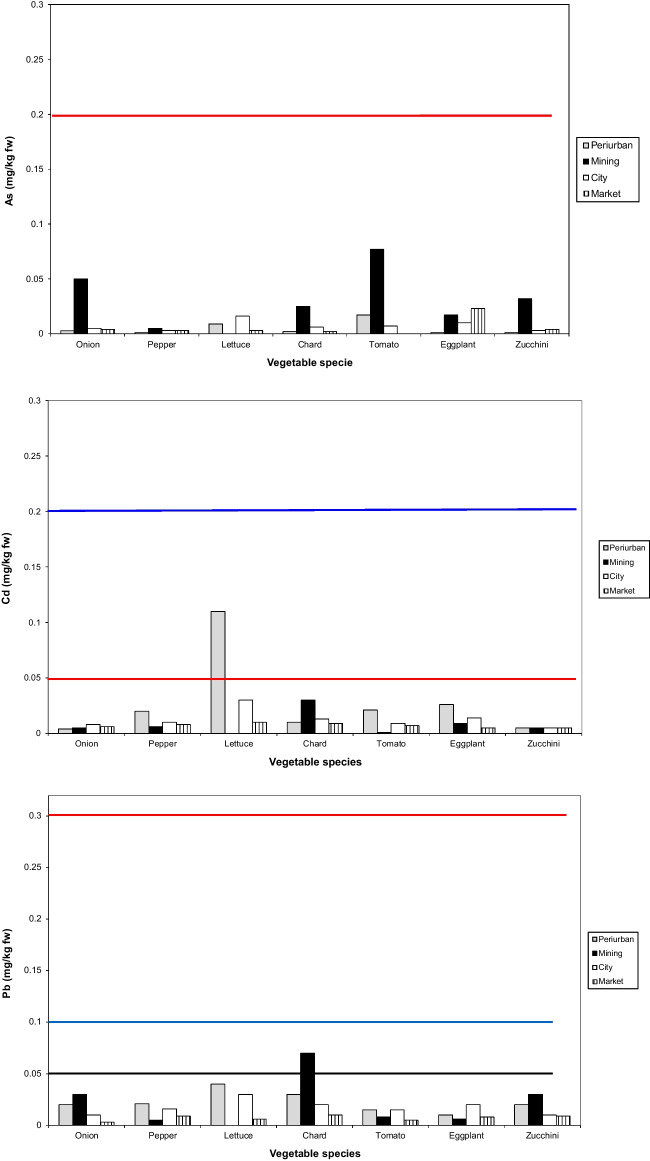
Mean value of As, Cd, and Pb concentration found in the different vegetable species cultivated in the urban gardens. For As, red line represents the maximum permissible concentration in food. For Cd, blue line represents the maximum permissible concentration for leafy species and red one for fruiting and bulbous vegetables. For Pb, black line is the maximum permissible concentration for fruiting species, blue line for bulbous species, and red line for leafy species

The mean concentration (fresh weight) of As, Cd and Pb in all the studied vegetable is shown in Fig. 4. All values were below the maximum permissible concentrations established by European legislation. The calculated hazard quotients (Table 4) showed that the estimates of parameters for non-carcinogenic risk were < 1 for all elements in all the studied vegetables growing in the different urban garden areas. HI values were also < 1 for all vegetables.

**Table 4:** Hazard quotient and hazard index (HI) of potentially toxic elements from studied food species cultivated in urban gardens. *M*, mining area; *PeriU*, periurban area

Elements	Chard			Onion			Zucchini			Tomato			Eggplant			Lettuce		Pepper		
	M	City	PeriU	M	City	PeriU	M	City	PeriU	M	City	PeriU	M	City	PeriU	City	PeriU	M	City	PeriU
As	5.44E-03	1.38E-03	5.64E-04	4.11 E-03	4.28E-04	2.31E-04	7.63E-03	6.76-E04	2.65E-04	7.77E-03	7.09E-04	1.63E-03	3.73E-03	2.24E-03	2.37E-04	7.00E-03	4.01E-03	1.66E-03	1.01E-03	3.74E-04
B	1.17E-03	8.83E-04	1.02E-03	1.86 E-03	1.30E-03	1.65E-03	1.01E-03	6.97-E04	1.10E-03	2.16E-03	1.65E-03	1.71E-03	4.74E-04	5.32E-04	6.69E-04	1.62E-03	2.76E-03	5.09E-04	5.37E-04	4.96E-04
Ba	6.37E-04	8.91E-04	2.45E-03	5.70±E-03	1.02E-03	3.85E-04	7.76E-05	1.31-E04	1.26E-04	1.17E-04	1.38E-04	1.50E-04	3.04E-05	4.25E-05	4.29E-05	9.20E-05	3.87E-04	2.86E-05	4.19E-05	4.17E-05
Cd	1.74E-03	8.50E-04	7.39E-04	1.16E-03	2.06E-03	9.66E-04	3.68-E04	3.38-E04	3.77E-04	3.11E-03	2.92E-03	6.89E-03	6.03E-04	4.67E-03	1.60E-03	3.73E-03	1.46E-02	5.55E-04	9.15E-03	1.89E-03
Co	2.37E-03	3.83E-03	2.28E-03	6.43E-03	6.78E-03	6.49E-03	2.45E-03	4.36E-03	2.85E-03	4.00E-03	1.37E-02	1.88E-02	3.87E-04	2.41E-03	2.36E-03	6.96E-03	4.34E-03	1.33E-03	4.45E-03	7.86E-03
Cr	8.14E-04	9.60E-04	3.75E-04	2.55E-03	3.82E-03	7.79E-04	6.63E-04	1.34E-03	2.93E-04	2.29E-03	2.40E-03	1.03E-03	3.35E-04	3.97E-04	2.13E-04	2.71E-03	1.26E-03	4.83E-04	9.05E-04	3.75-E04
Cu	1.64E-03	1.54E-03	1.36E-03	4.54E-03	3.57E-03	3.39E-03	3.05E-03	2.00E-03	2.20E-03	8.22E-03	5.91E-03	7.08E-03	1.30E-03	1.31E-03	2.10E-03	2.83E-03	3.76E-03	1.69E-03	1.94E-03	2.57E-03
Mo	1.36E-03	1.34E-03	4.80E-04	8.67E-03	3.91E-03	996E-04	3.03E-03	2.44E-03	3.19E-03	4.89E-03	5.19E-03	4.82E-03	5.02E-04	7.82E-04	1.01E-03	2.40E-03	5.31E-04	4.69E-04	7.17E-04	2.36E-04
Ni	1.80E-04	1.36E-04	6.10E-05	3.68E-03	7,74E-04	3.28E-04	1.60E-04	5.21E-04	7.90E-05	4.15E-04	6.39E-03	2.43E-04	8.55E-05	5.88E-05	5.42E-05	3.14E-04	1.69E-04	1.56E-04	1.92E-04	1.33E-04
Pb	2.09E-03	6.50E-04	8.33E-04	3.32E-03	1,69E-03	2.75E-03	1.78-E03	5.77E-04	7.91E-04	1.35E-03	2.49E-03	2.51E-03	1.89E-04	6.59E-04	4.19E-04	2.07E-03	2.69E-03	2.22E-04	7.06E-04	9.22E-04
Zn	1.06E-03	6.81E-04	7.78E-04	2.02E-03	1.75E-03	2.75E-03	1.68-E03	1.10E-03	1.98E-03	3.35E-03	2.03E-03	2.90E-03	4.31E-04	64.54E-04	7.94E-03	1.84E-03	3.23E-03	4.59E-04	5.92E-04	8.45E-04
HI	**0.018**	**0.013**	**0.011**	**0.036**	**0.027**	**0.021**	**0.022**	**0.014**	**0.013**	**0.038**	**0.044**	**0.048**	**0.008**	**0.014**	**0.009**	**0.032**	**0.038**	**0.008**	**0.012**	**0.016**

**Fig. 5:**
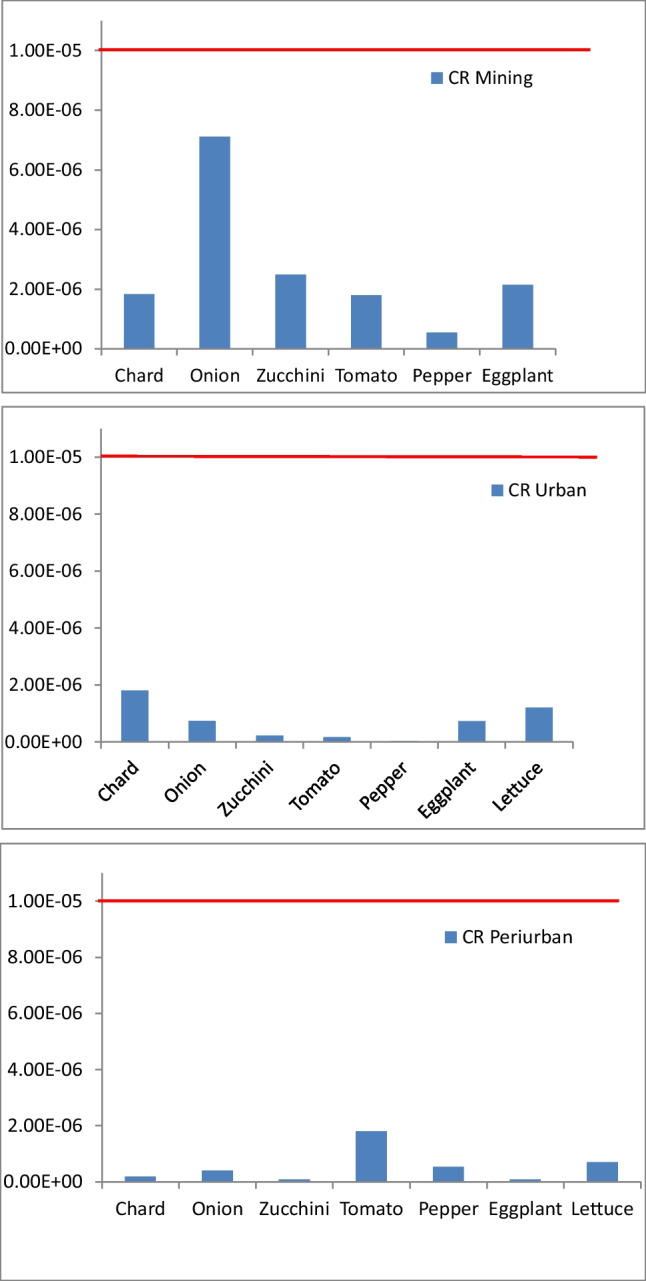
Cancer risk of As in the studied vegetables cultivated in different urban gardens from mining, city and peri-urban area. Red line is the permissible value

Also, in the scenario of haze, values of HQ were below 1 for lettuce and chard (0.043 and 0.014 respectively). The values of CR of As (Fig. 5) for the studied species were below the limit (10^−5^) in the three studied areas and also during the haze period for chard and lettuce (7.67E − 07 and 1.80E − 06 respectively).

## Discussion

### Soil contamination

The concentrations of As, Cr, Cu, Ni, Pb, and Zn in the cities and peri-urban garden soils were relatively low taking as reference the Maximum Permissible Concentrations (MAC) (Kabata-Pendias and Pendias 2011), which are the values most commonly reported in the literature for trace metals in agricultural soils. The maximum values indicated by Kabata-Pendias and Pendias (2011) were not exceeded by the concentrations found in city and peri-urban orchards (Table 1), except for Cu in ALC, Ni in ALA, MI2, TOR, and LEV. Although the concentrations of PTEs in soils of city and peri-urban orchards remained relatively low, soils from the city had higher levels of As, Pb, and Zn. The PLI and CF values indicate the absence of contamination (PLI < 1) in the peri-urban area (UTR orchard) as well as in some city orchards (LUC, TRI, GUA, ELE, HER, and TOR). All these orchards are located in the metropolitan area of Seville. But even in this group, in the ALC orchard, Cu CF exceeded the value of 3, being 3 the limit between moderate and considerable contamination. Copper CF peaks with values close to 3 were also observed in the ASO, LEV, and MOR orchards and somewhat lower in the ALA, HER, and MI2 orchards (Fig. 1). This Cu contamination was possibly produced by copper fungicides, which are products authorized by European organic farming regulations (European Commission 2008) and which might be used regularly and even indiscriminately by non-professional horticulturists in these orchards. This fact has been observed by López et al. (2019) in another urban garden of Seville city.

In some urban orchards (ALA and MI2 in Seville city, ASO and LEV in Cordoba city), high PLI values were observed (PLI 1.5–1.6), due to the increase of Pb CF (considerable contamination) but also due to As and Cu CFs (moderate contamination) (Fig. 1). A wide variability in Pb levels in the city orchards was found (range 13.4–92.9 mg kg^−1^, Table 1). Hiller et al. (2016), in a study with urban soils, also found greater variability in Pb contents than for other contaminating metals (such as Cu, Zn, and Hg). Lead contamination in soils has been a problem in numerous cases from very different possible sources (Pelfrêne et al. 2019; Romero-Baena et al. 2021). One of the most common origins has been the historical contamination by leaded gasoline, which may have affected the urban gardens. The trigger action value (TAV) indicated by the regional regulations for Pb is 275 mg kg^−1^ (Junta de Andalucia 2015), but all soil Pb concentrations found in city and peri-urban locations were below TAV. Concentrations registered for As and Cu in peri-urban and city orchards (with maximum values of 19.7 mg kg^−1^ for As and 265 mg kg^−1^ for Cu) were lower than the TAVs indicated in the regional standard (36 and 595 mg kg^−1^ respectively) (Junta de Andalucía 2015).

In the case of urban city soils, their pH was alkaline and the high pH would a priori promote the immobilization of the soil PTE, limiting the transfer to plants.

PTE concentrations in gardens located in the mining area (NER and TIN) were much higher than in the rest of the city and peri-urban gardens (Table 1), except in the case of Ni concentrations, which were relatively similar in all cases. Kabata-Pendias and Pendias (2011) also published trigger action values (TAV) for trace metals in agricultural soils from various reports, documents, and internet data in some European countries. In the case of NER and TIN, the average or maximum concentrations (Table 1) exceeded the reported TAV for some elements such as As (65 mg kg^−1^) and Pb (300 mg kg^−1^) or were of similar magnitude (TAV Cu 500 mg kg^−1^, Zn 1500 mg kg^−1^). In NER and TIN gardens from the mining area, As concentration (48.9 and 85.5 mg kg^−1^) was also greater than the regional TAV for As (36 mg kg^−1^, Junta de Andalucia 2015) with maximum concentrations higher than values reported by Romero-Baena et al. (2021). In the case of Pb, the regional TAV (295 mg kg^−1^) was exceeded in the orchards from the mining area. The maximum Pb values reported in this study were much higher than concentrations reported in soil family gardens from the same mining area (Romero-Baena et al. 2021). The PLI in the mining orchards (Fig. 1) showed a significant average contamination. However, the contamination was very high (6 < CF < 12) when considering the CFs of Pb, As, and Cu, as observed in Fig. 1. In the case of Cr, its concentration was much higher in the mining orchards than in the other ones (Table 1) with maximum values higher than concentrations reported by Romero-Baena et al. (2021), but it was always below the regional TAV (10,000 mg kg^−1^). In fact, the CFs of Cr in the NER and TIN soils (Fig. 1) were only slightly higher than 1 (1.1 and 1.3 respectively) indicating that the higher concentration of this element, in the mining area, was due to the soil parent rocks.

These results were consistent with the long history of mining activity in the area (NER, TIN), focused on the exploitation of sulfuric, Cu, Zn, Pb, Ag, and Au from pyrite and polymetallic deposits and from oxidized or gossan mineralization. These soils had neutral pH values (Table 1). Other authors (Fernández-Caliani et al. 2009; Monaci et al. 2011) reported acid pH for these soils with values sometimes below 4, but Romero-Baena et al. (2021) reported pH values close to 6 in soils from family gardens of the mining area. The addition of organic amendments and limestone, which are practices frequently carried out by horticulturists, may have increased pH values. Results suggest that preventive measures to ensure safer gardening include actions to limit the direct ingestion of contaminated soil particles such as washing hands accurately after gardening and that liming practices must be carried out before soils are used for crop cultivation.

#### Potentially toxic element levels in vegetables and plant/soil relation

Values of PTEs varied mainly with the plant species and to a lesser extent with the sampling sites. Results from plant analysis indicate that anthropogenic contamination related to the city and mining activities increases human health risks associated with urban agriculture only for some elements. Arsenic was high in vegetables cultivated in urban gardens from the mining area, reflecting the high As concentration in these soils. Onion and chard were the species with the highest mean As concentration (0.558 and 0.272 mg kg^−1^ respectively). The mean concentrations of As in all the studied vegetables (Fig. 4) were below the maximum permissible concentration established by the European Directive (EC 2023). This indicates that bioavailable As was also depending on soil properties such as organic matter, the content of Fe and Al, and bacterial activities which may control its plant availability (Kabata-Pendias and Pendias 2011; Paltseva et al. 2018).

According to the European Directive (EC 2021), Cd concentration in leafy, fruiting, and bulbous vegetables should be lower than 0.20, 0.05, and 0.05 mg kg^−1^ (fresh weight basis, fw) respectively.

Cadmium in all vegetables was below the permissible limits, similar to results reported by Varol et al. (2022) in vegetables from Turkey. Only in one case did lettuce from the peri-urban garden slightly exceed the guidance value (0.20 mg kg^−1^ fw). The high Cd content in some vegetables (pepper, eggplant, tomato, lettuce) found in peri-urban areas compared with the other areas might be due to particular pollution sources in that area. Antisari et al. (2015) found a peak in leaf Cd concentration in several species from a control-rural site and attributed it to long-term soil fertilization which built up Cd in soils, especially phosphate fertilizer and also pesticides (Hua et al. 2022). In our case, the peri-urban orchard had a long history as an olive grove receiving mineral fertilization for many years. Values of Cd in soils from the peri-urban area are not available since it was not possible to measure soil Cd with the technique (pXRF) used because it was below the detection limit. In any case, values of Cd found in this study were lower than values reported in leafy species grown in peri-urban gardens from contaminated sites in Uganda (Nabulo et al. 2012). Lead concentration was high in mining soils, but only some vegetables (chard, onion, and zucchini) cultivated in this area reflected higher Pb concentrations than those from other urban gardens. It is interesting to note that in peri-urban areas, the Pb content in all crops was similar to the one found in city gardens, even if these soils had the lowest Pb content. This can be due to the low correlation between Pb content in soil and vegetables (Säumel et al. 2012; McBridge et al. 2014; Romero-Baena et al. 2021), but can also suggest that other anthropic sources of Pb pollution (probably atmospheric) might contribute to Pb contents in vegetables as reported by other authors (Säumel et al. 2012; McBridge et al. 2014). None of the Pb values exceeded the permissible value established by the European Directive (EC 2023) for leafy, bulbous, and fruiting vegetables (0.3, 0.1, and 0.05 mg kg^−1^ fw respectively), which might be due to the neutral pH of these soils. The highest Pb contents were found in chard from the mining area (0.16 mg kg^−1^ fw) and in lettuce from the MI2 city garden (0.10 mg kg^−1^ fw). In the mining area, a great portion of As, Cu, Pb, and Zn should be immobilized in the organic matter (Romero-Baena et al. 2021) reducing the bioavailability of plants. Generally, in the family garden soils of the Riotinto mining area, the bioavailability of PTEs is low (Romero-Baena et al. 2021).

There are no data about the recommended maximum Ba level permitted in food, and Kabata-Pendias and Pendias (2001) reported a range of 2–13 mg kg^−1^. Only chard showed high values in all areas (maximum content in chard from peri-urban garden and Huelva city, 82 and 67 mg kg^−1^ respectively), and such contents were lower than in leafy species cultivated in New York City (McBridge et al. 2014). Cetin and Jawed (2024) reported that Ba concentration in the leaf of ornamental species is related to traffic density, and there is no correlation between total soil Ba and crop Ba (McBridge et al. 2014). It looks like that chard has a good efficiency to take up Ba from the growth environment.

The normal range of Cr in vegetables is 0.04–0.13 mg kg^−1^ (Kabata-Pendias and Pendias 2001), and almost all vegetables from gardens of the four areas exceeded these amounts such as Mo average (normal range 0.1–0.8) and B (normal range 6–14 mg kg^−1^). The highest Cr content was observed in lettuce and onion from urban gardens of Seville (2.75 mg kg^−1^ in MI2 and 2.62 mg kg^−1^ in ALA respectively). These values were much higher than Cr concentrations found in lettuce of Bratislava city (Hiller et al. 2022) but lower than the maximum values found in other leafy species of Berlin city (Säumel et al. 2012). Chromium is not an essential element for plants, and the high Cr concentration in plants was not related to its concentration in soils, but some other atmospheric sources might be responsible.

Molybdenum concentration in plants reflects the soluble Mo pool in soils (Kabata-Pendias and Pendias 2001). The maximum Mo content was reported for lettuce from Huelva city (MOR, 28.1 mg kg^−1^). We have no data on Mo in soils but industrial pollution or agricultural practices may be responsible for the high values recorded in plants. The reported concentration for Ni and Zn in vegetables is 0.06–3 and 10–73 mg kg^−1^ respectively (Kabata-Pendias and Pendias 2001), and in general, the studied vegetables from all the studied areas did not exceed this range (with the only exception of zucchini from peri-urban area).

The mean Cu content in the studied vegetables was in a range of 4.34–17.9 mg kg^−1^, exceeding only a few cases the values of 10 mg kg^−1^ reported for vegetables (Kabata-Pendias and Pendias 2001). The highest value was found in chard from an urban garden of Seville city (M12, 79 mg kg^−1^), higher than the one reported for the same species in Berlin city (Säumel et al. 2012). As reported above, Cu treatments are permitted in ecological agriculture for pest control.

In our study, only a few crops (mainly lettuce and chard) from city urban gardens had PTE contents higher than samples from the local market, in contrast to results reported for the city of Berlin (Sämuel et al. 2012), which underlines the security of urban horticulture in the city areas. Food species cultivated in the city gardens also have in general the same PTE concentrations as vegetables harvested in peri-urban sites, and more differences were related to the species. In the present study, leafy species generally accumulated more PTEs in comparison with fruiting species, except for Mo and Ni. Similar findings have also been reported in other studies (Zhuang et al. 2017; Rossini-Oliva and López 2021; Hiller et al. 2022; Varol et al. 2022). Bulbous and leafy species have the same capacity to accumulate As and Cr. Pelfrêne et al. (2019) showed that fresh herbs had the highest values of Pb and Cd while fruiting vegetables presented the lowest contents of both elements. Values of TF (Fig. 3) indicated that these elements were not accumulated in the edible part of these species in spite of their concentration in soils. The TF ratio may quantify the potential health risk associated with vegetable consumption (Boim et al. 2016). All values were lower than 1 indicating a general low risk because of their relatively low accumulation in edible parts. The transfer factors (TF) varied depending on the plant species and sometimes may also vary depending on the area studied (Fig. 3). It is interesting to point out that in the mining area where As and Pb concentration in urban garden soils is high, onion was the species with the highest As TF, and onions and chard showed the highest Pb TF.

Haze can increase atmospheric pollution by enriching with different PTEs such as As, Cr, or Cu (Luo et al. 2016). In leafy vegetables, an increase of PTE (As, Co, Cr, Cu, Fe, and Ni) was observed after the haze episode due mainly to dust particles coming from the Sahara desert. Especially during these conditions, it is very important to wash edible parts of vegetables before eating.

### Human health risk ingestion assessment

Carcinogenic risk values, associated exclusively with exposure to As, were not higher than 10^−5^ in all vegetables cultivated in urban garden from the studied area (Fig. 5). As the safe limit was not overpassed, therefore, its ingestion should not represent a risk to human health. Despite the high values of some PTE found in the studied vegetables, the HQ and HI indexes were below 1 for all studied vegetables, including after the haze episode. Similar results were reported by Varol et al. (2022) for several vegetables grown in Turkey and by Margenat et al. (2019) for vegetables cultivated in Spain but contrary to the results reported by Shaheen et al. (2016) for fruits and vegetables in Bangladesh. The highest values of HI were observed for tomato, onion, and lettuce. These findings are consistent with previous studies (Margenat et al. 2019). Cancer risk values varied mainly with the site and not according to the species. In conclusion, there is no carcinogenic health risk by consuming these plant foods individually and collectively through the diet of the seven vegetable species, and therefore, the ingestion of vegetables from the tested gardens is safe for humans. Results suggest also that the total concentration of PTEs in the soil is not a good indicator to a priori establish risks since it can overestimate human risks. This is very important since on many occasions, data without a correct risk assessment procedure can create social alarms. In addition, it is very important to share information with gardeners and local authorities on both the benefits and the risks associated with urban gardening activities and vegetable species that should accumulate more PTEs.

## Conclusions

The concentration of PTEs in vegetables is related to the species and urban gardens where they are grown. Soils of orchards from mining areas are more contaminated than city and peri-urban ones except for Ni. There was a lack of correlation between vegetable and paired soil concentrations for almost all studied elements. The level of PTEs in vegetables was in general similar to market and peri-urban concentrations and differences were related to the species. Mining activities and traffic can increase PTEs in crops. Arsenic, Cd, and Pb did not exceed the guidance values for foods. Chromium, Mo, and Ba concentrations were high in many vegetables from all the studied areas and should be monitored more often. In spite of that, the hazard quotient for all the studied elements and hazard index in all vegetables were below the guidance values. Cancer risk values for As were below the established limits for all the studied vegetables, including in the mining area, which values indicate that there is no cancer risk by its ingestion. Therefore, the consumption of urban garden produce should not present a significant health hazard. During haze episodes, an increase of some PTEs can occur in vegetables, so it is very important to wash them accurately.

### Supplementary Information

Below is the link to the electronic supplementary material.

Supplementary file1 (DOCX 98.5 KB)

Supplementary file2 (DOCX 15.9 KB)
