# Supplementary material for: Is it healthy urban agriculture? Human exposure to potentially toxic elements in urban gardens from Andalusia, Spain
Source: Environ Sci Pollut Res Int. 2024 May 15;31(25):36626–42. doi: 10.1007/s11356-024-33500-w (PMC11182867; doi:10.1007/s11356-024-33500-w)
Supplement: Supplementary file 1 — Supplementary file1 (DOCX 98.5 KB) [file 11356_2024_33500_MOESM1_ESM.docx]

**Cr**

**Chard**

**Chard**

**Chard**

**Chard**

**Co**

**Ni**

**As**


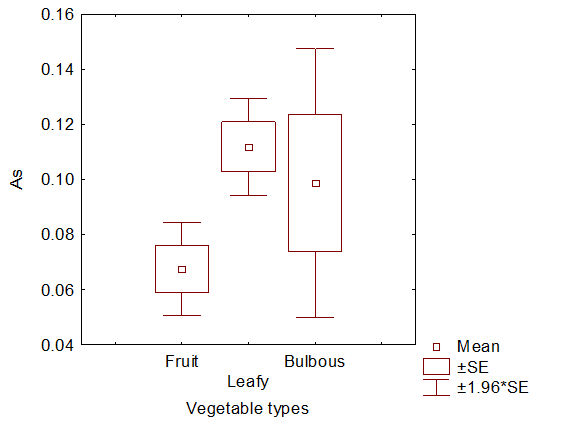


**Lettuce**

**Fe**

**Cu**

**Chard**

Figure 1. Mean concentration (mg kg-1) of PTE in chard and lettuce 
during haze and not haze periods.
