# Supplementary material for: Is it healthy urban agriculture? Human exposure to potentially toxic elements in urban gardens from Andalusia, Spain
Source: Environ Sci Pollut Res Int. 2024 May 15;31(25):36626–42. doi: 10.1007/s11356-024-33500-w (PMC11182867; doi:10.1007/s11356-024-33500-w)
Supplement: Supplementary file 2 — Supplementary file2 (DOCX 15.9 KB) [file 11356_2024_33500_MOESM2_ESM.docx]

Table 1. Summary of the studied urban gardens

| Acronym | Garden name | City | Area^1^ | Coordinate 1 | Coordinate 2 | Foundation  (year) | Nearby relevant facilities |  | |  |
| --- | --- | --- | --- | --- | --- | --- | --- | --- | --- | --- |
| GUA | Guadaira | Seville | U | 37°21'18.6"N | 5°58'05.9"W | 2016 | High traffic road at 50m, on the city edge |  | |  |
| ALA | Alamillo | Seville | U | 37°24'44.0"N | 5°59'50.2"W | 2013 | High traffic road at 20-150 m, on the city edge |  | |  |
| ALC | Alcosa II | Seville | U | 37°24'57.5"N | 5°55'12.9"W | 2008 | Highway 50m, on the city edge |  | |  |
| HER | Hercules | Seville | U | 37°19'41.7"N | 5°57'52.8"W | 2010 | Railway track at 30 m, on the city edge |  | |  |
| TRI | Vega de Triana | Seville | U | 37°23'04.6"N | 6°01'02.2"W | 2015 | ON the city edge |  | |  |
| MI2 | Miraflores II | Seville | U | 37°25'22.7"N | 5°57'05.1"W | 2010 | High traffic road at 20-200 m, railway track at 20 m, on the city edge |  | |  |
| ELE | Infanta Elena | Seville | U | 37°23'56.4"N | 5°55'57.2"W | 2021 |  |  | |  |
| TOR | Torreblanca | Seville | U | 37°23'27.4"N | 5°54'25.6"W | 2021 | On the city edge |  | |  |
| LUC | Santa Lucía | Alcala de Guadaira | U | 37°19'45.4"N | 5°49'38.9"W | 2019 | On the city edge |  | |  |
| ASO | Asomadilla | Cordoba | U | 37°54'03.9"N | 4°46'47.8"W | 2011 | On the city edge |  | |  |
| LEV | Hortecor and Levante | Cordoba | U | 37°51'48.5"N | 4°47'09.4"W | 2022 | Close to the city centre |  | |  |
| MOR | Moret | Huelva | U | 37°16'25.2"N | 6°56'13.6"W | 2010 | Inside the main city park of Huelva |  | |  |
| UHU | Campus Univ. | Huelva | U | 37°26'17.2"N | 6°92'15.6"W | 2010 | Inside the University of Huelva, close to the motorway |  | |  |
| NER | Urbano | Nerva | M | 37°41'34.5"N | 6°32'36.1"W |  | Surrounded by mines less than 1 km away |  | |  |
| TIN | Alto de la Mesa | Riotinto | M | 37°41'42.0"N | 6°35'20.7"W | 2022 | Big mine at 100 m |  | |  |
| UTR | Los Adrianes | Utrera | PU | 37°13'22.5"N | 5°46'14.8"W | 2017 | Agricultural use |  | |  |
| ^1^ U: urban, PU: periurban, M: mining | | | | | | | | |  |  |
